# Increased nuclear suppressor of cytokine signaling 1 in asthmatic bronchial epithelium suppresses rhinovirus induction of innate interferons

**DOI:** 10.1016/j.jaci.2014.11.039

**Published:** 2015-07

**Authors:** Vera Gielen, Annemarie Sykes, Jie Zhu, Brian Chan, Jonathan Macintyre, Nicolas Regamey, Elisabeth Kieninger, Atul Gupta, Amelia Shoemark, Cara Bossley, Jane Davies, Sejal Saglani, Patrick Walker, Sandra E. Nicholson, Alexander H. Dalpke, Onn-Min Kon, Andrew Bush, Sebastian L. Johnston, Michael R. Edwards

**Affiliations:** a Airway Disease Infection Section, National Heart and Lung Institute, Imperial College London, London, United Kingdom; f Respiratory Pediatrics, National Heart and Lung Institute, Imperial College London, London, United Kingdom; c Centre for Respiratory Infection, Imperial College London, London, United Kingdom; b MRC & Asthma UK Centre in Allergic Mechanisms of Asthma, London, United Kingdom; d Imperial College Healthcare National Health Service Trust, London, United Kingdom; e Pediatric Medicine, University of Bern, Bern, Switzerland; g Department of Infectious Diseases, Medical Microbiology and Hygiene, University of Heidelberg, Heidelberg, Germany; h Walter & Eliza Hall Institute, Parkville, Australia; i Department of Medical Biology of the University of Melbourne, Parkville, Australia

**Keywords:** Rhinovirus, asthma, asthma exacerbation, atopy, interferon, innate immunity, cytokine, T_H_2 inflammation, suppressor of cytokine signaling, AA, Atopic asthma, BAL, Bronchoalveolar lavage, BEC, Bronchial epithelial cell, CISH, Cytokine-inducible SH2-containing protein, GFP, Green fluorescent protein, ISG, Interferon-stimulated gene, ISRE, Interferon-stimulated response element, KC, Keratinocyte-derived chemokine, LIX, LPS-induced CXC chemokine, NANA, Nonatopic nonasthmatic, NF-κB, Nuclear factor κB, NLS, Nuclear localization sequence, polyI:C, Polyinosinic-polycytidylic acid, SOCS, Suppressor of cytokine signaling, SOCS1wt, Full-length wild-type human SOCS1, STAT, Signal transducer and activator of transcription, STRA, Severe therapy-resistant atopic asthma

## Abstract

**Background:**

Rhinovirus infections are the dominant cause of asthma exacerbations, and deficient virus induction of IFN-α/β/λ in asthmatic patients is important in asthma exacerbation pathogenesis. Mechanisms causing this interferon deficiency in asthmatic patients are unknown.

**Objective:**

We sought to investigate the expression of suppressor of cytokine signaling (SOCS) 1 in tissues from asthmatic patients and its possible role in impaired virus-induced interferon induction in these patients.

**Methods:**

We assessed *SOCS1* mRNA and protein levels *in vitro*, bronchial biopsy specimens, and mice. The role of SOCS1 was inferred by proof-of-concept studies using overexpression with reporter genes and *SOCS1*-deficient mice. A nuclear role of SOCS1 was shown by using bronchial biopsy staining, overexpression of mutant SOCS1 constructs, and confocal microscopy. SOCS1 levels were also correlated with asthma-related clinical outcomes.

**Results:**

We report induction of SOCS1 in bronchial epithelial cells (BECs) by asthma exacerbation–related cytokines and by rhinovirus infection *in vitro*. We found that SOCS1 was increased *in vivo* in bronchial epithelium and related to asthma severity. *SOCS1* expression was also increased in primary BECs from asthmatic patients *ex vivo* and was related to interferon deficiency and increased viral replication. In primary human epithelium, mouse lung macrophages, and *SOCS1*-deficient mice, SOCS1 suppressed rhinovirus induction of interferons. Suppression of virus-induced interferon levels was dependent on SOCS1 nuclear translocation but independent of proteasomal degradation of transcription factors. Nuclear SOCS1 levels were also increased in BECs from asthmatic patients.

**Conclusion:**

We describe a novel mechanism explaining interferon deficiency in asthmatic patients through a novel nuclear function of SOCS1 and identify SOCS1 as an important therapeutic target for asthma exacerbations.

Asthma exacerbations are the major cause of morbidity, mortality, and health care costs in asthmatic patients and cause a decrease in lung function.^1^ Respiratory tract virus infections, of which human rhinoviruses are by far the most common,^2,3^ cause the great majority of asthma exacerbations. The mechanisms involved in asthma exacerbations are poorly understood, but increased susceptibility to rhinovirus infections is strongly implicated.^4,5^

We originally reported impaired induction of the innate antiviral IFN-β^6^ and IFN-λ^7^ by rhinovirus infection in lung cells from asthmatic patients and implicated deficiency of IFN-λ in asthma exacerbation severity in human subjects *in vivo*.^7^ Recent studies have confirmed deficient respiratory tract virus induction of IFN-α, IFN-β, and/or IFN-λ in bronchial epithelial cells (BECs), bronchoalveolar lavage (BAL) macrophages, peripheral blood dendritic cells, and PBMCs from asthmatic patients.^8-14^ Although impaired interferon induction might be associated with asthma control,^15^ the mechanism or mechanisms responsible for impaired interferon induction are currently unknown. Two recent studies reported that exogenous TGF-β enhanced rhinovirus replication in fibroblasts and BECs and that this was accompanied by reduced interferon levels.^16,17^ The latter study also reported that anti–TGF-β treatment of BECs from asthmatic patients was accompanied by reduced suppressor of cytokine signaling *(SOCS) 1* and *SOCS3* gene expression,^17^ possibly associating these SOCS proteins with interferon deficiency, but no investigations of SOCS function were performed.

There are 7 SOCS family members in human subjects and mice: SOCS1 through SOCS6 and cytokine-inducible SH2-containing protein (CISH). The family is characterized by a central SH2 domain and a C-terminal SOCS box motif that couples SOCS proteins to a Cullin-RING E3 ubiquitin ligase complex. Therefore SOCS proteins can act as adaptors to target bound proteins for ubiquitination and proteasomal degradation and thus function as negative regulators of cytokine signaling. SOCS1 through SOCS3 have been studied in detail, including development of knockout mice.^18-20^ *SOCS1* deletion causes fatal inflammation, which can be rescued by deletion of *IFNG*.^18^ In mice SOCS1 and SOCS2 negatively regulate T_H_2 immunity^19,21-23^; however, a human polymorphism enhancing SOCS1 expression is associated with asthma.^24^ T-cell SOCS3 mRNA levels are increased in asthmatic patients and correlate with IgE levels,^20^ but a functional role for SOCS3 in human asthma is unknown, and thus the role of SOCS proteins in asthma is unclear.

In the context of viral infections, SOCS proteins suppress cytokine receptor signaling through inhibition of Janus-activated kinase and signal transducer and activator of transcription (STAT) signaling,^25-27^ and preliminary data suggest that SOCS1 and SOCS3 might suppress influenza-induced IFN-β promoter activation.^28^ However, there are no data on the possible role of SOCS proteins in suppressing viral induction of interferons in patients with asthma and during asthma exacerbations.

We hypothesized that SOCS1/3 would be induced by proinflammatory cytokines and rhinovirus infection in BECs *in vitro*. Thus we investigated SOCS expression in human primary BECs from asthmatic patients *ex vivo* and their possible role in interferon deficiency and increased viral replication in these cells. We also investigated whether SOCS1/3 proteins could directly suppress viral induction of innate interferons in airway cells *in vitro* and *in vivo*. We found that SOCS1, but not SOCS3, levels were increased in cells from asthmatic patients and also found that nuclear localization of SOCS1 was required for suppression of virus-induced interferons. This suppression was independent of the only known nuclear function of SOCS1, which is induction of proteasomal degradation of signaling proteins. Thus we describe a novel mechanism explaining interferon deficiency in asthmatic patients, a new nuclear function of SOCS1, and identify SOCS1 as an important therapeutic target for asthma exacerbations.

## Methods

For detailed methods, including patient data, animal models, reagents, experimental protocols, and statistical analysis, please see the Methods section and Tables E1-E3 in this article's Online Repository at www.jacionline.org.

## Results

### SOCS1 is induced in primary BECs by proinflammatory cytokines and rhinovirus

**Fig E1:**
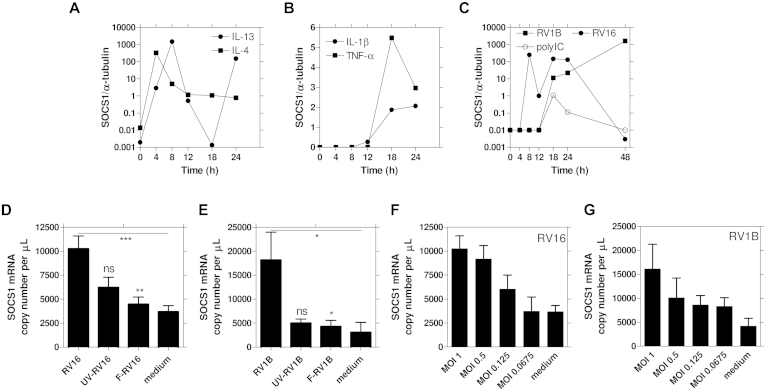
Densitometry of SOCS1 Western blots in Fig 1, effects of SOCS1 mRNA induction by rhinovirus after UV inactivation and filtration, and dose-dependent induction of SOCS1 mRNA by rhinovirus. **A-C,** Primary human BECs were treated with IL-4 or IL-13 (Fig E1, *A*) or TNF-α or IL-1β (Fig E1, *B*) or were infected with RV1B, RV16, or polyI:C (1 μg/mL; Fig E1, *C*), and total protein was harvested over time and plotted versus α-tubulin as a control protein (n = 1 experiment for all induced SOCS1 protein). **D** and **E,** BECs were infected with RV16 (Fig E1, *D*), UV-inactivated RV16, or filtered RV16, or RV1B (Fig E1, *E*). *F-RVIB*, Filtered RV1B; *UV-RV1B*, UV-inactivated RV1B. UV-inactivated and rhinovirus-filtered (virus-free) preparations both showed a reduction in SOCS1 mRNA expression. **F** and **G,** BECs infected with RV16 (Fig E1, *F*) or RV1B (Fig E1, *G*) showed dose-dependent inductions of SOCS1 mRNA. *P < .05, **P < .01, and ***P < .001, as indicated or versus rhinovirus treatment, by means of 1-way ANOVA. *ns*, Not significant. There were 3 to 4 experiments from BECs derived from 3 to 4 healthy control donors.

**Fig 1:**
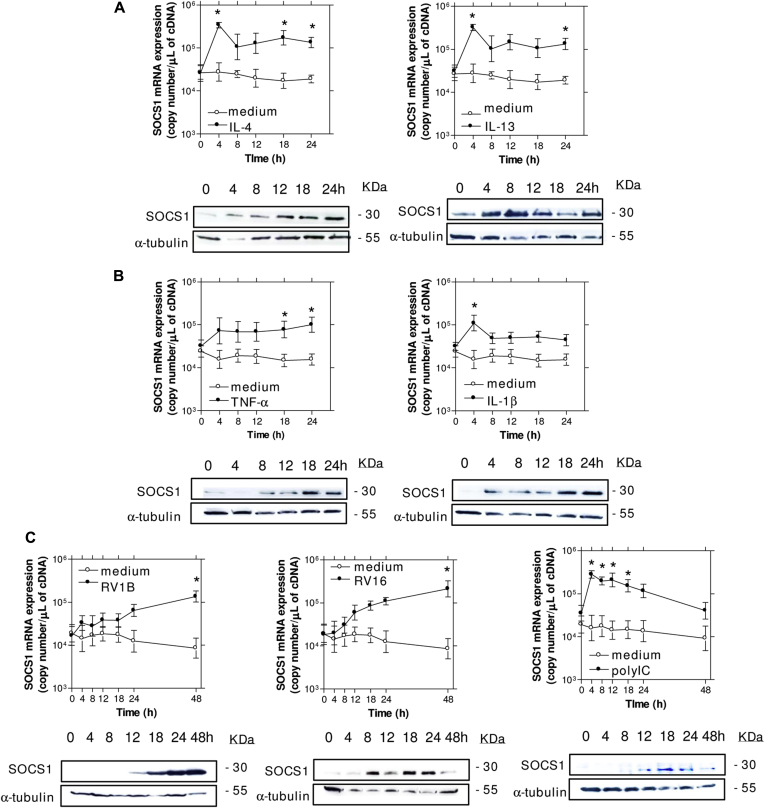
SOCS1 mRNA and protein were induced in primary BECs by viruses and cytokines important in asthma pathogenesis. **A,** The T_H_2 cytokines IL-4 and IL-13 both induced SOCS1 mRNA and protein in a time-dependent manner. **B,** The proinflammatory cytokines TNF-α and IL-1β also induced SOCS1 mRNA and protein in a time-dependent manner. **C,** RV1B, RV16, and 1 μg/mL polyI:C all induced SOCS1 mRNA and protein in a time-dependent manner. *P < .05 versus medium-treated cells.

*SOCS3* mRNA expression is increased in T cells in asthmatic patients,^20^ but upregulation of SOCS1 by IL-13 in airway smooth muscle cells from asthmatic patients is impaired.^22^ Thus whether SOCS proteins are upregulated in asthmatic patients is uncertain, and whether SOCS proteins are upregulated in cells that are infected by respiratory tract viruses is unknown. Therefore we first investigated the effects of the T_H_2 cytokines IL-4 and IL-13 on *SOCS1* through *SOCS6* and *CISH* mRNA and protein expression in BECs because these cytokines are strongly implicated in asthma pathogenesis.^29,30^ IL-4 and IL-13 both induced *SOCS1* mRNA and protein expression (Fig 1, *A*). Densitometric analysis for the Western blots in Fig 1 are shown in Fig E1 in this article's Online Repository at www.jacionline.org. No other SOCS proteins/mRNAs were induced by IL-4 or IL-13, with the exception of CISH, which was significantly induced by both.

**Fig E2:**
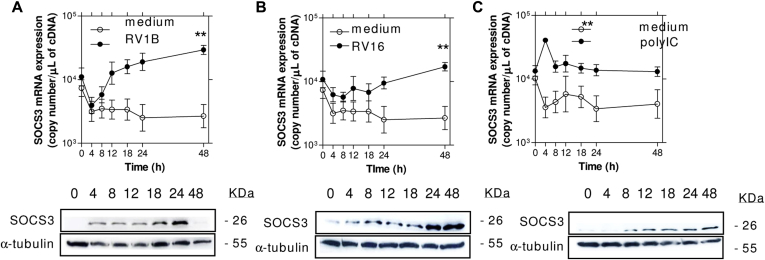
SOCS3 mRNA and protein were induced in BECs by rhinovirus and polyI:C. Primary human BECs were infected with RV1B, RV16, or polyI:C (1 μg/mL), and total RNA and total protein were harvested over time. RV1B **(A)**, RV16 **(B)**, and polyI:C **(C)** all induced SOCS3 mRNA and protein in a time-dependent manner. Values are presented as means ± SEMs (n = 4 experiments). **P < .01 versus medium treatment, 2-way ANOVA.

We next investigated the ability of the proinflammatory cytokines TNF-α and IL-1β, rhinovirus infection, and polyinosinic-polycytidylic acid (polyI:C; as a mimic of other viral infections) to induce SOCS expression in BECs. We found that TNF-α and IL-1β both induced SOCS1 (Fig 1, *B*) but not any other SOCS family member, whereas both SOCS1 (Fig 1, *C*) and SOCS3 (see Fig E2 in this article's Online Repository at www.jacionline.org) were induced by RV1B (representative of minor group rhinoviruses), RV16 (major group), and polyI:C. RV1B and RV16 did not induce SOCS2, SOCS4 through SOCS6, or CISH in BECs. The induction of *SOCS1* by RV1B and RV16 was susceptible to UV irradiation and through filtering out virus with a 30-kDa molecular weight filter and was dose dependent (see Fig E1). These data indicate that SOCS1 is induced by proinflammatory cytokines and rhinovirus infection in primary human BECs.

### SOCS1 protein expression is increased in bronchial epithelium from asthmatic adults

**Fig 2:**
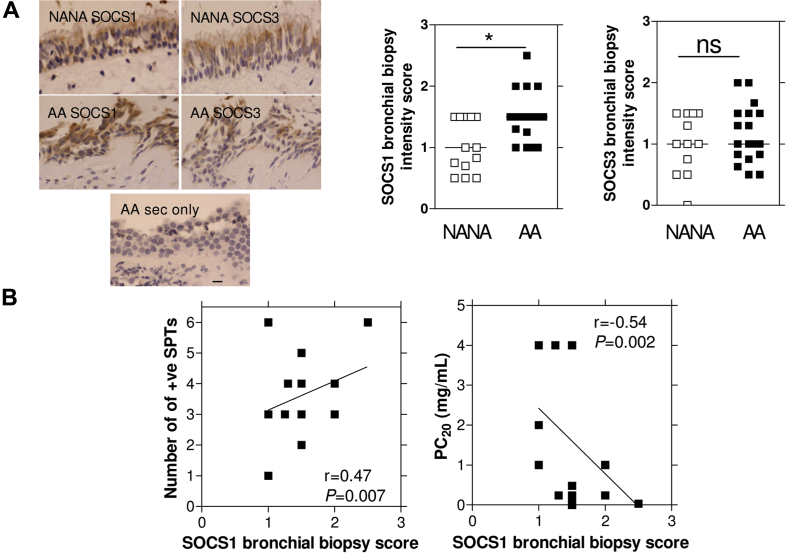
SOCS1 protein levels were increased in bronchial biopsy specimens from adults with mild-to-moderate AA compared with those seen in NANA adults and correlated with asthma-related clinical outcomes. **A,** Representative pictures showing epithelial staining of SOCS1 *(left panels)* and SOCS3 *(right panels)*. *Bar* = 10-μm scale (×40 objective was used in all pictures). Patients with AA showed significantly more SOCS1, but not SOCS3, staining compared with that seen in NANA subjects. *P < .05, *bar* represents median. *ns*, Not significant. **B,** SOCS1 bronchial biopsy scores positively correlated with the number of positive skin pick test responses *(SPTs)* and negatively correlated with the dose of histamine causing a 20% reduction in lung function (PC_20_).

We next investigated the abundance of SOCS1 and SOCS3 proteins in biopsy specimens of adults with uninfected mild-to-moderate atopic asthma (AA) compared with nonatopic nonasthmatic (NANA) adult control donors. SOCS1, but not SOCS3, staining intensity was significantly increased in the bronchial epithelium of patients with AA compared with that seen in healthy NANA subjects (Fig 2, *A*). There was a positive correlation between SOCS1 staining scores and numbers of positive skin prick test responses, with a similar nonsignificant trend for IgE levels (data not shown) and a negative correlation with the provocative concentration of histamine causing a 20% reduction in lung function (PC_20_ histamine), indicating greater intensity of SOCS1 staining was related to greater severity of atopy and airway hyperresponsiveness (Fig 2, *B*). In contrast, SOCS3 biopsy staining scores did not significantly correlate with any clinical outcome. SOC1 protein expression did not correlate with numbers of exacerbations (data not shown).

### SOCS1 completely suppressed interferon promoter activation in BECs

Having established that SOCS1 levels are increased in patients with AA and related to airway hyperresponsiveness to histamine, the ability of SOCS1 to modulate rhinovirus induction of interferons in BECs *in vitro* was examined. We focused our attention on induction of IFN-β and IFN-λs because these are the interferon subtypes induced by viral infection of BECs.^31^ Because total interferon induction is a consequence of both direct viral induction of interferon and subsequent paracrine interferon induction of interferon, we investigated both viral and interferon induction of the IFN-β and IFN-λ1 promoters, as well as interferon induction of promoters of interferon-responsive genes.

**Fig E3:**
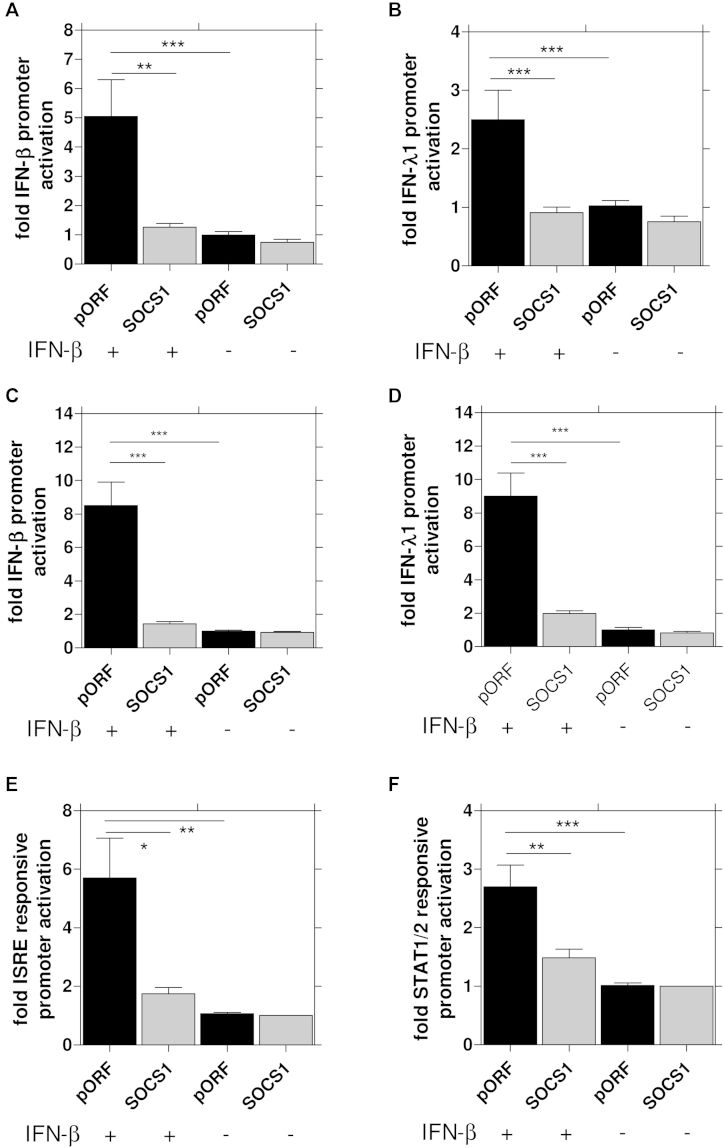
SOCS1 overexpression inhibited IFN-β–induced IFN-β and IFN-λ1 promoter activation and activation of minimal promoters containing ISRE and STAT1/2 binding sites. Human BECs were transfected with plasmids encoding SOCS1 or empty vector pORF. **A** and **B**, IFN-β (5 ng/mL) induced IFN-β promoter activation (Fig E3, *A*) and IFN-λ1 promoter activation (Fig E3, *B*) at 24 hours, which were both significantly suppressed by SOCS1 (n = 4 experiments). BEAS-2B cells were transfected with plasmids encoding SOCS1 or empty vector pORF. SOCS1 also inhibited both IFN-β (5 ng/mL)–induced IFN-β promoter activation (Fig E3, *C*) and IFN-λ1 promoter activation (Fig E3, *D*) at 24 hours. **E** and **F**, SOCS1 also inhibited IFN-β–induced ISRE-driven promoter activation (Fig E3, *E*) and STAT1/2-driven promoter activation (Fig E3, *F*) at 24 hours. Values are presented as means ± SEMs (n = 3 experiments). *P < .05, **P < .01, and ***P < .001, as indicated, by using 1-way ANOVA with the Bonferroni multiple comparison test.

We found that overexpression of SOCS1 in both primary human BECs and in the human BEC cell line BEAS-2B (see Fig E3 in this article's Online Repository at www.jacionline.org) completely inhibited exogenous IFN-β–induced activation of both the IFN-β and IFN-λ1 promoters. In BEAS-2B cells SOCS1 also suppressed interferon induction of a minimal promoter containing the interferon-stimulated response element (ISRE) and a minimal promoter containing a STAT1/2-responsive element (see Fig E3), which are type I interferon–responsive promoters induced by the interferon-stimulated gene factor 3 and STAT1/2 transcription factor complexes, respectively, and are typical readouts for interferon signaling.

**Fig E4:**
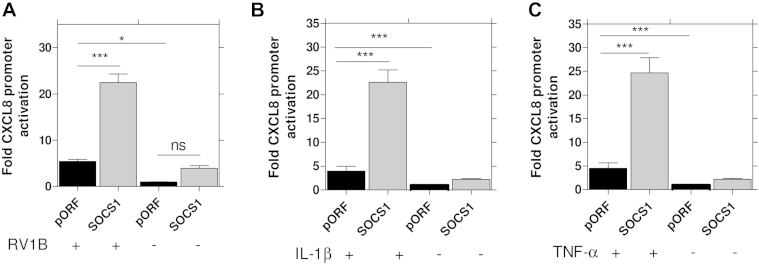
SOCS1 overexpression increased RV1B-, IL-1β– and TNF-α–induced CXCL8 promoter activation. BEAS-2B cells were seeded and transfected with SOCS1 or the empty vector control pORF and infected with RV1B or treated with 10 ng/mL IL-1β or TNF-α. SOCS1 overexpression increased RV1B-induced **(A)**, IL-1β–induced **(B)**, and TNF-α–induced **(C)** CXCL8 promoter activation at 24 hours. Values are presented as means ± SEMs (n = 4 experiments). *P < .05 and ***P < .001, 1-way ANOVA with the Bonferroni multiple comparison test. *ns*, Not significant.

**Fig 3:**
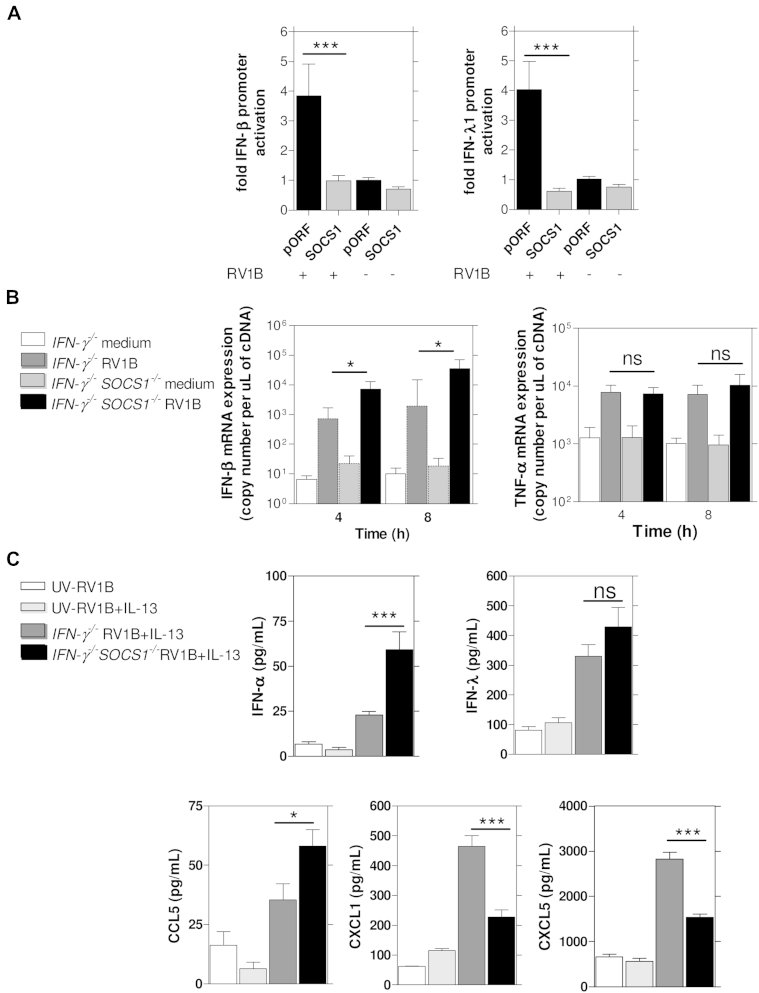
SOCS1 suppressed rhinovirus-induced interferon induction but not rhinovirus-induced proinflammatory cytokine induction. **A,** SOCS1-transfected cells showed completely suppressed RV1B-induced IFN-β and IFN-λ1 promoter activation versus pORF empty vector control at 24 hours. ***P < .001. **B,** RV1B-induced IFN-β mRNA expression was increased in *ex vivo*–cultured BAL macrophages from *SOCS1*^*−/−*^*IFN-γ*^*−/−*^ mice compared with *IFN-γ*^*−/−*^ mice. No differences were observed between these 2 groups for RV1B-induced TNF-α mRNA. *P < .05. **C,** RV1B-induced IFN-α expression (8 hours after infection) was significantly increased in RV1B-infected *SOCS1*^*−/−*^*IFN-γ*^*−/−*^ mice compared with *IFN-γ*^*−/−*^ mice. BAL IFN-λ (24 hours) levels showed a nonsignificant trend for increase in RV1B-infected *SOCS1*^*−/−*^*IFN-γ*^*−/−*^ mice, whereas CCL5 levels (24 hours) were also significantly increased in RV1B-infected *SOCS1*^*−/−*^*IFN-γ*^*−/−*^ mice compared with *IFN-γ*^*−/−*^ mice. CXCL1/KC and LIX/CXCL5 (both 48 hours) were both decreased in BAL fluid from RV1B-infected *SOCS1*^*−/−*^*IFN-γ*^*−/−*^ mice compared with *IFN-γ*^*−/−*^ mice. A mixture of *SOCS1*^*−/−*^*IFN-γ*^*−/−*^ and *IFN-γ*^*−/−*^ mice was used for the UV-RV1B and UV-RV1B plus IL-13 groups. *P < .05 and ***P < .001. *ns*, Not significant.

Overexpression of SOCS1 also completely suppressed rhinovirus-induced IFN-β and IFN-λ1 promoter activation in primary human BECs (Fig 3, *A*). In contrast, overexpression of SOCS1 in BEAS-2B cells significantly increased rhinovirus-, IL-1β–, and TNF-α–induced CXCL8 promoter activation (around 20- to 25-fold; see Fig E4 in this article's Online Repository at www.jacionline.org).

### Augmented IFN-β expression in BAL macrophages from *SOCS1*-deficient mice

To determine whether the converse were true, namely whether the absence of *SOCS1* would lead to augmentation of interferon induction, we used *ex vivo*–cultured BAL macrophages from *SOCS1*^*−/−*^*IFN-γ*^*−/−*^ mice and control *IFN-γ*^*−/−*^ mice and found that in the absence of *SOCS1*, *IFN-β* mRNA induction by rhinovirus at 4 and 8 hours was significantly increased compared with that seen in *IFN-γ*^*−/−*^ control mice (Fig 3, *B*). This enhancement was specific to interferon induction because BAL macrophages from *SOCS1*^*−/−*^*IFN-γ*^*−/−*^ mice and *IFN-γ*^*−/−*^ mice showed no difference in induction of TNF-α mRNA by rhinovirus (Fig 3, *B*).

### Induction of SOCS1 inhibited interferon induction *in vivo*

**Fig E5:**
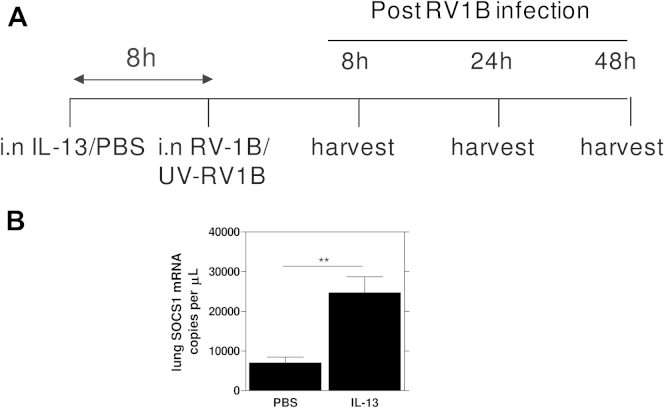
IL-13 treatment increased lung SOCS1 expression. *IFN-γ*^*−/−*^ C57Bl/6 mice were treated with 0.5 μg of IL-13 or PBS (vehicle) through the intranasal *(i.n.)* route. **A,** Schematic diagram showing the timing of IL-13/PBS treatment and RV1B/UV-RV1B infection 8 hours later. BAL and lung mRNA was harvested at 8, 24, and 48 hours after infection. **B,** IL-13 treatment induced SOCS1 mRNA in *IFN-γ*^*−/−*^ mice at 4 hours after IL-13 treatment compared with PBS-treated mice. Values are presented as means ± SEMs (n = 4 animals per group). **P < .01, *t* test.

We next investigated the importance of SOCS1 in regulating rhinovirus-induced interferon *in vivo* using *IFN-γ*^*−/−*^ and *SOCS1*^*−/−*^*IFN-γ*^*−/−*^ mice. Mice were pretreated with IL-13 for 8 hours to enhance SOCS1 levels before rhinovirus infection (see Fig E5, *A*, in this article's Online Repository at www.jacionline.org). IL-13 pretreatment significantly enhanced SOCS1 mRNA expression in the lungs of *IFN-γ*^*−/−*^ mice by approximately 3-fold (see Fig E5, *B*). As expected, there was no SOCS1 expression in SOCS1-deficient mice. On rhinovirus infection, IL-13–pretreated *IFN-γ*^*−/−*^ mice in which SOCS1 was induced had significantly deficient IFN-α, trends toward deficient IFN-λ, and significantly deficient RANTES/CCL5 (an interferon-inducible chemokine) in BAL fluid when compared with IL-13–pretreated *SOCS1*^*−/−*^*IFN-γ*^*−/−*^ mice, in which SOCS1 could not be induced (Fig 3, *C*). Consistent with our observation that enhanced SOCS1 expression substantially enhanced rhinovirus induction of the CXCL8 promoter in human BECs *in vitro* (see Fig E4, *A*), enhanced SOCS1 expression significantly augmented rhinovirus induction of the mouse CXCL8 homologues keratinocyte-derived chemokine (KC)/CXCL1 and LPS-induced CXC chemokine (LIX)/CXCL5 *in vivo* (Fig 3, *C*).

### Increased SOCS1 levels in BECs from asthmatic children were associated with interferon deficiency

**Fig 4:**
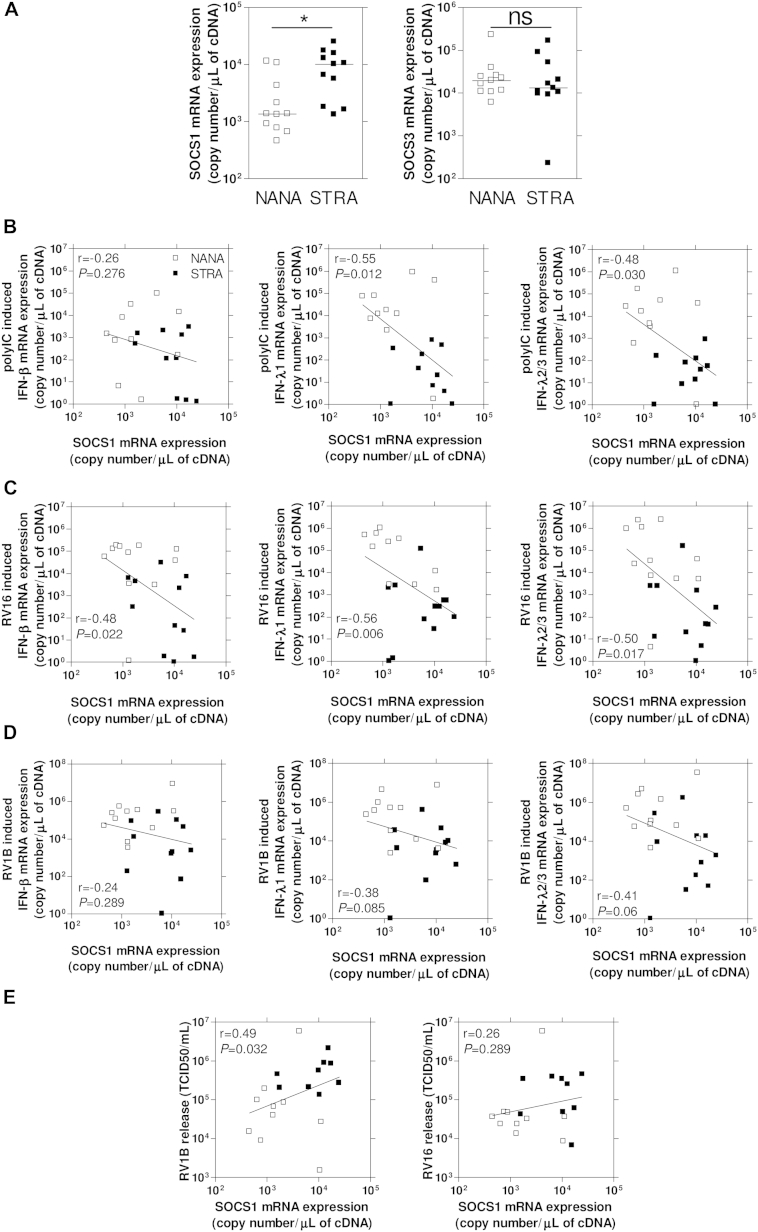
SOCS1, but not SOCS3, mRNA expression was increased in primary BECs from children with severe asthma compared with that seen in control children and was related to impaired interferon induction and increased viral release. **A,** SOCS1 mRNA levels were increased at baseline in children with STRA compared with NANA subjects. No differences between NANA subjects and children with STRA were observed for SOCS3 mRNA levels. *P < .05. *ns*, Not significant. **B,** PolyI:C induced IFN-β, IFN-λ1, and IFNλ2/3 mRNA levels 8 hours after treatment negatively correlated with baseline SOCS1 mRNA levels. **C,** RV16-induced IFN-β/λ1/λ2/3 mRNA levels 24 hours after infection negatively correlated with baseline SOCS1 mRNA levels. **D,** RV1B-induced IFN-β/λ1/λ2/3 mRNA levels 24 hours after infection showed trends toward a negative correlation with baseline SOCS1 mRNA levels. **E,** RV16 and RV1B release 48 hours after infection, as measured by means of titration in HeLa cells, positively correlated with baseline SOCS1 mRNA levels.

BECs from children with STRA with confirmed rhinovirus-induced interferon deficiency^14^ were used to investigate whether SOCS1 levels are increased in primary BECs from patients with severe asthma. We also sought to establish whether there were relationships between SOCS1 levels, interferon deficiency, and viral replication. *SOCS1*, but not *SOCS3*, mRNA expression levels were increased (approximately 8- to 9-fold) in unstimulated and uninfected primary human BECs from children with severe asthma compared with those in BECs from NANA control subjects (Fig 4, *A*). *SOCS1* mRNA levels in the unstimulated and uninfected cells were significantly inversely correlated with *IFN-λ1* and *IFN-λ2/3* mRNA induction by polyI:C, with a similar but nonsignificant trend for *IFN-β* (Fig 4, *B*) and, importantly, with induction of all 3 interferon subtypes by RV16 (Fig 4, *C*). However, RV1B showed no significant correlation (Fig 4, *D*). Baseline *SOCS1* mRNA levels correlated positively with RV1B release at 48 hours after infection in BECs but did not correlate with RV16 release (Fig 4, *E*).

### SOCS1 suppression of interferons required SOCS1 nuclear translocation

**Fig E6:**
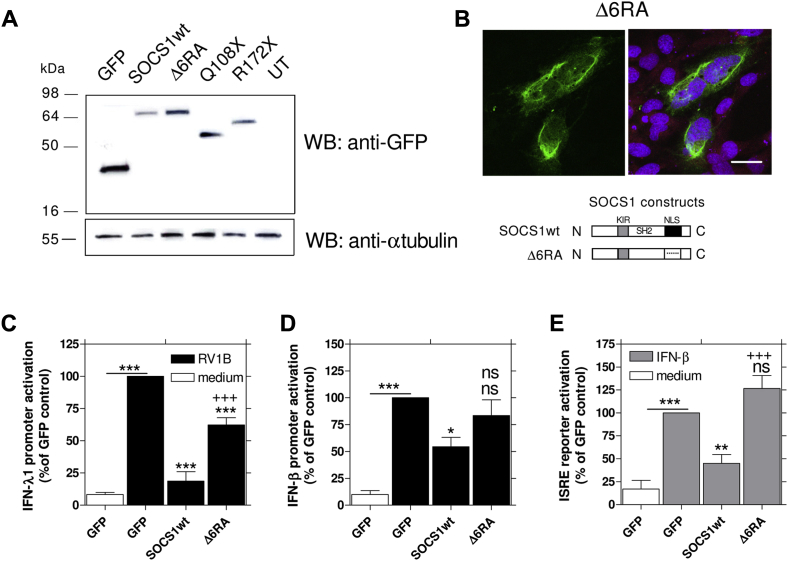
Mutation of the SOCS1 NLS resulted in reduced suppression of rhinovirus-induced interferon promoter and IFN-β–induced minimal ISRE responsive promoter activation compared with SOCS1wt. **A**, All SOCS1 mutants expressed equally as assessed by anti-GFP detection when compared with anti–α-tubulin using Western blotting *(WB)*. *UT*, Untransfected. **B**, The SOCS1 NLS mutant Δ6RA showed predominately cytoplasmic localization by using confocal microscopy. *Left panel*, GFP; *right panel*, 4′-6-Diamidino-2-phenylindole dihydrochloride. Evans blue and GFP overlay: *bar* = 10-μm scale. A diagram of SOCS1wt and the NLS mutant Δ6RA shows the 6 mutated amino acid residues in the NLS region. **C**, Δ6RA showed less suppression compared with SOCS1wt of RV1B-induced IFN-λ1. **D**, RV1B-induced IFN-β promoter activation. **E**, IFN-β (5 ng/mL) induced ISRE minimal promoter activation at 24 hours (n = 4 independent experiments). *P < .05, **P < .01, and ***P < .01, as indicated and versus RV1B-infected or IFN-β–treated GFP-transfected cells. +++*P* < .001 versus SOCS1wt-transfected, RV1B-infected, or IFN-β–treated cells by using 1-way ANOVA with the Bonferroni multiple comparison test. *ns*, Not significant (*upper ns* vs SOCS1wt, RV1B-infected, or IFN-β–treated cells; *lower ns* vs GFP, RV1B-infected, or IFN-β–treated cells).

**Fig 5:**
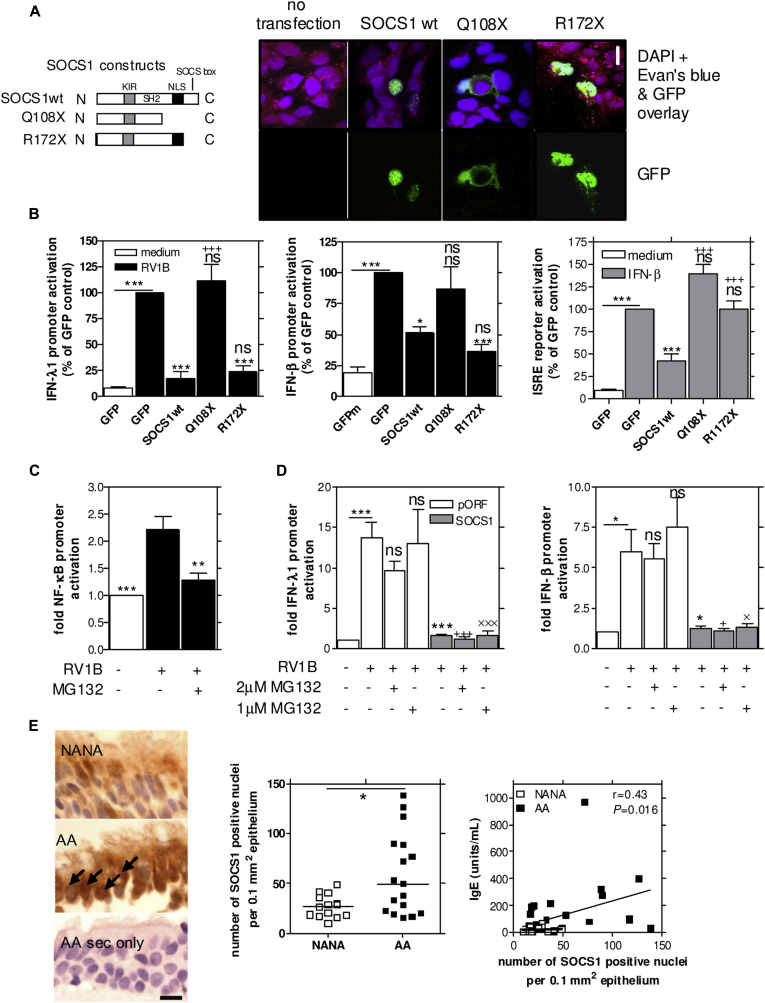
SOCS1-mediated suppression of rhinovirus-induced interferon expression required nuclear translocation but not proteasome-mediated degradation. **A,** Confocal microscopy showed nuclear localization of SOCS1wt and the R172X mutant, whereas the Q108X mutant showed only cytoplasmic localization. All images used the ×60 objective. *Bar* = 10-μm scale. *DAPI*, 4′-6-Diamidino-2-phenylindole dihydrochloride. **B,** SOCS1wt and R172X both suppressed RV1B-induced interferon promoter activation, whereas Q108X did not. SOCS1wt, but neither Q108X nor R172X, suppressed IFN-β–induced minimal ISRE-responsive promoter activation. *P < .05 and ***P < .001, as indicated and versus GFP empty vector–transfected, RV1B-infected, or IFN-β–treated group. +++*P* < .001 versus SOCS1wt-transfected rhinovirus-infected or IFN-β–treated cells. *ns*, Not significant (*upper ns*, not significant vs SOCS1wt, RV1B-infected, or IFN-β–treated cells; *lower ns*, not significant vs GFP, RV1B-infected, or IFN-β–treated cells). **C,** MG132 inhibited RV1B-induced NF-κB activation. **P < .01 and ***P < .001 versus the RV1B-infected untreated group. **D,** MG132 had no effect on SOCS1-mediated suppression of rhinovirus-induced IFN-λ1 or IFN-β promoter activation. *P < .05 and ***P < .001, as indicated and versus the pORF-transfected RV1B-infected untreated group. +*P* < .05 and +++*P* < .001 versus the pORF-transfected RV1B-infected group treated with 2 μmol/L MG132. ×*P* < .05 and ×××*P* < .001 versus the pORF-transfected RV1B-infected group treated with 1 μmol/L MG132. *ns*, Not significant versus the pORF-transfected RV1B-infected untreated group. **E,** Increased nuclear SOCS1 expression in BECs was observed in patients with AA compared with that seen in NANA subjects, and nuclear SOCS1 staining correlated with IgE levels in these subjects. All images used the ×60 objective. *Black arrows* indicate nuclear SOCS1 staining. *Bar* = 10-μm scale. *P < .05.

SOCS1 can prevent nuclear factor κB (NF-κB) signaling by entering the nucleus through a C-terminal proximal nuclear localization sequence (NLS) and targeting NF-κB p65 for proteasomal degradation through the C-terminal SOCS box.^32^ Therefore we hypothesized that SOCS1 might suppress rhinovirus-induced interferon induction by translocating into the nucleus and initiating proteasomal degradation of transcription factors required for interferon induction. To investigate the role of nuclear translocation of SOCS1 and of the SOCS box, we used vectors expressing green fluorescent protein (GFP)–tagged full-length wild-type human SOCS1 (SOCS1wt) and 2 mutants. The mutants included SOCS1 truncations with both the NLS and the SOCS box deleted (Q108X) or with a deleted SOCS box alone, leaving the NLS intact (R172X; Fig 5, *A*).^32^ We found that the SOCS1 mutant that lacked the NLS (Q108X) was indeed unable to translocate to the nucleus; however, both SOCS1wt and R172X, which had a deleted SOCS box but intact NLS, were able to translocate to the nucleus (Fig 5, *A*). We then tested the ability of these constructs to suppress rhinovirus induction of interferons in BEAS-2B cells and found that the construct lacking the NLS (Q108X) had lost its ability to suppress rhinovirus-induced IFN-β and IFN-λ promoter activation, whereas fully intact SOCS1 (SOCS1wt containing both the NLS and the SOCS box) and R172X (containing the NLS but lacking the SOCS box) were still suppressive (Fig 5, *B*). Furthermore, SOCS1wt, but neither Q108X nor R172X, suppressed interferon-induced ISRE promoter activation. This definitively proves that SOCS1-mediated suppression of rhinovirus-induced interferon is NLS dependent but SOCS box independent and therefore distinct from interferon-induced ISRE activation, which is dependent on both the NLS and the SOCS box (Fig 5, *B*). Furthermore, the requirement for nuclear localization for both rhinovirus- and interferon-induced responses was supported with a full-length SOCS1 construct containing mutated NLS residues (Δ6RA), which was impaired in its ability to enter the nucleus and exhibited a less suppressive effect on interferon induction when compared with SOCS1wt (see Fig E6, *B-D*, in this article's Online Repository at www.jacionline.org). SOCS1wt, R172X, and Q108X proteins were expressed at similar levels, as determined by using Western blotting (see Fig E6, *A*).

Because the construct (R172X) lacking the SOCS box (which is required for initiation of proteasomal degradation) still suppressed rhinovirus-induced interferon promoter activation (Fig 5, *B*), this suggested that SOCS1-mediated suppression of rhinovirus-induced interferon was independent of proteasomal degradation. Therefore we next investigated whether pretreatment with the 28S proteasome inhibitor MG132 would be able to prevent SOCS1-mediated inhibition of rhinovirus-induced interferon. At a concentration of 1 μmol/L, MG132 significantly suppressed rhinovirus-induced NF-κB promoter activation, which is dependent on proteasomal degradation of IκB and therefore sensitive to this inhibitor (Fig 5, *C*). We found that neither the 1 μmol/L dose nor the 2 μmol/L dose had any effect on SOCS1-mediated suppression of rhinovirus-induced IFN-β or IFN-λ promoter activation, confirming that proteasomal degradation is not involved in SOCS1-mediated suppression of rhinovirus-induced interferon induction (Fig 5, *D*).

Finally, to determine whether nuclear SOCS1 levels were increased in asthmatic patients, we re-evaluated protein staining for SOCS1 in bronchial biopsy specimens from patients with mild-to-moderate AA and nonatopic healthy subjects and specifically assessed only nuclear staining. Nuclear SOCS1 staining was clearly observed in the BEC layer in these biopsy specimens, with significantly higher levels of SOCS1 nuclear staining in patients with AA compared with healthy subjects (Fig 5, *E*). Furthermore, numbers of nuclear SOCS1-positive cells positively correlated with serum total IgE levels in these subjects. Nuclear SOCS1 levels did not correlate with exacerbation numbers (data not shown).

## Discussion

Impaired interferon induction in response to rhinovirus and other viral infections *ex vivo* has been reported recently and in several studies is related to markers of underlying asthma severity.^6,7,11,13,33^ Furthermore, trends toward a higher viral load in asthmatic patients compared with that seen in healthy control subjects have been observed *in vivo*.^5^ The mechanism or mechanisms responsible for this impaired induction of interferon are unknown. Here we describe increased expression of SOCS1 in asthmatic patients, the importance of its nuclear rather than cytoplasmic function, and its role in deficient interferon induction. These data together identify avenues to inhibit the expression or function of SOCS1 as potential therapies for asthma exacerbations, boosting deficient interferon responses and potentially suppressing harmful inflammatory chemokine induction.

Previous studies of SOCS1 expression in asthmatic patients have led to contradictory findings.^22,24^ The induction and role of SOCS1 in airway epithelium has been poorly studied to date, with a single study reporting increased *SOCS1* mRNA expression in response to IFN-γ stimulation of primary BECs.^34^ We found that a number of stimuli increased *SOCS1* mRNA expression, including T_H_2 and proinflammatory cytokine levels, rhinovirus, and polyI:C. Uninfected (stable) patients with AA had increased SOCS1 protein staining in bronchial biopsy specimens compared with nonasthmatic subjects, which we argue was likely a result of ongoing allergic airways inflammation. The observed increased expression in patients with stable asthma would mean that on viral infection, the ability to respond with rapid interferon induction would be impaired. This is entirely in keeping with the delayed and quantitatively impaired early interferon induction reported in studies identifying interferon deficiency in asthmatic patients.^6,13,14^

We further found that SOCS1 expression was induced by exacerbation-related and virus-induced proinflammatory cytokines, polyI:C, and rhinovirus infection of BECs. This strongly supports the idea that SOCS1 expression is likely to be further upregulated as asthma exacerbations progress, which is consistent with observations of substantially impaired interferon responses and greater viral replication in lung cells at later time points,^6,7,13,14^ increased duration of rhinovirus-related lower airways symptoms in asthmatic patients,^4^ and strong relationships between impaired interferon induction and asthma exacerbation severity *in vivo*.^7^

In cell lines SOCS1 can suppress interferon induction by influenza viruses.^28^ In the present study we only investigated SOCS1 expression and the role of SOCS1 in rhinovirus infection. Although rhinovirus is the main trigger of asthma exacerbations, other viruses can cause asthma exacerbations, and whether this is in part due to impaired antiviral immunity in lung epithelial cells remains unclear. Therefore we cannot claim that the role of SOCS1 in suppressing virus-induced interferon levels is limited to rhinovirus infection. It would be of interest to the field to examine the role of SOCS1 in other respiratory tract virus infections; of interest, impaired interferon induction has been observed in PBMCs and dendritic cells from patients with respiratory syncytial virus and influenza, respectively.^8,9^ Recently, Spann et al^35^ showed higher viral loads in respiratory syncytial virus– and metapneumovirus-infected tracheal epithelial cells from wheezy children, but no impairments in type or type III interferons were observed.^35^ Clearly, more studies are required to determine whether impaired interferon induction is mostly associated with rhinovirus infection and whether SOCS1 can impair interferon induction by other respiratory tract viruses in primary BEC *ex vivo* models. Therefore our data potentially explain why interferon is impaired in asthmatic patients but does not explain why rhinovirus is the most frequent cause of asthma exacerbations.

The increased SOCS1 protein levels correlated with clinical markers of asthma (PC_20_) and also numbers of positive skin prick test responses, suggesting a relationship between SOCS1 expression and AA. At this point, we cannot definitively conclude whether SOCS1 expression is increased because of asthma, atopy, or both. Furthermore, because our study numbers remain small, there is a need for further studies with larger patient numbers to confirm whether SOCS1 expression is related to clinical markers of asthma or atopy. We speculated that bronchial epithelial SOCS1 expression might be increased because of ongoing airway inflammation, and our findings that SOCS1 expression was induced by T_H_2 and non-T_H_2 cytokines support this hypothesis. Because the non-T_H_2 cytokines TNF-α and IL-1β also induced SOCS1, this is unlikely to be a strictly T_H_2-dependent process. However, the link between SOCS1 and T_H_2 responses has been previously established. SOCS1 is a negative regulator of T_H_2 responses.^21,23^ SOCS1 has a known role in hematopoietic cells. Increased SOCS1 levels in hematopoietic cells act to counter excessive T_H_2 outgrowth, whereby in BECs excessive T_H_2 cytokine signaling might also induce SOCS1, but our results suggest this likely hampers epithelium-derived innate interferon induction and immunity to viruses. Indeed, we found that bronchial epithelial SOCS1 expression correlated with the number of positive skin prick test responses and airway hyperresponsiveness but not exacerbation numbers, suggesting that SOCS1 can be increased in response to but not limited to allergic inflammation. In support, Baraldo et al^11^ have shown a clear association between impaired rhinovirus-induced interferon induction in asthmatic patients and increased T_H_2 cytokine expression in the bronchial mucosa. The antagonistic effects of interferons on T_H_2 signaling and the allergic cascade and *vice versa* is also underscored by other studies.^9,12,36-39^ Further studies are required to see whether impaired interferon induction is consistent with other markers of non-T_H_2 inflammation. Therefore we argue that therapies reducing T_H_2, TNF, or IL-1β signaling could enhance antiviral immunity in asthmatic patients. The latter hypothesis is supported by the observed therapeutic effect in selected populations that anti-T_H_2 therapies have recently been shown to have on asthma exacerbations.^29,30,40^ Although clinical studies investigating the effects of anti–IL-1β therapies are yet to be performed, the effects of TNF therapy on asthma exacerbation rates have been reported by just one study.^41^ Anti-TNF therapy (etanercept) had no effect versus placebo on the asthma exacerbation rate; however, this rate was extremely low across both groups (n = 1 each), and therefore no definitive conclusion can be reached. Considering the findings in this article, the effects of anti–IL-1β and anti-TNF therapy on interferon responses and asthma exacerbation rates and severity are warranted and would be of interest.

We found that SOCS1 suppressed virus-induced interferon induction but augmented IL-8/CXCL8 *in vitro* and augmented KC/CXCL1 and LIX/CXCL5 in mice *in vivo*. Having excess SOCS1 in BECs could be doubly deleterious in patients with asthma exacerbations in that beneficial antiviral pathways mediated by interferons are suppressed and harmful proinflammatory responses are augmented. These data clearly point to approaches that will inhibit the expression or function of SOCS1 as novel strategies for therapeutic intervention in patients with asthma exacerbations.

We found that SOCS1 was a potent suppressor of virus-induced type I and type III interferon induction. We originally attempted to grow tracheal epithelial cells from *SOCS1*^*−/−*^ mice but found these difficult to grow with established protocols. This omission is an unfortunate limitation of our study. We then opted to use BAL macrophages from *SOCS1*^*−/−*^ mice and found that *SOCS1*^*−/−*^ mice had enhanced interferon induction on rhinovirus infection compared with wild-type mice, enforcing the idea that SOCS1 is a negative regulator of virus-induced interferon. Consistent with the literature in other systems,^25,26^ we also found that SOCS1 regulated interferon-induced signaling in BECs. IFN-α/β/λs can all act as interferon-stimulated genes (ISGs), being induced by IFN-β and thus providing a positive feedback loop,^42,43^ and we observed that SOCS1 inhibited IFN-β–induced IFN-β and IFN-λ1 promoter activation. SOCS1-mediated inhibition of interferon receptor signaling has been shown to be dependent on the SOCS box.^26,44^ The SOCS box deletant R172X, which could undergo nuclear translocation, profoundly inhibited rhinovirus-induced IFN-β and IFN-λ promoter activation but not that of an ISRE-containing promoter, which is dependent on interferon receptor signaling, therefore proving that this SOCS1 mutant suppresses virus-induced rather than interferon-induced interferon induction. These data are consistent with the known requirement for the SOCS box in SOCS-mediated suppression of interferon receptor signaling.

In contrast, SOCS1 suppression of rhinovirus-induced interferon induction was independent of the SOCS box and did not require proteasomal degradation. SOCS1-mediated suppression of rhinovirus-induced interferon induction was very clearly dependent on nuclear localization because the SOCS1 construct unable to localize to the nucleus (Q108X) was unable to suppress rhinovirus-induced interferon promoter activation, and another construct with the NLS mutated (Δ6RA) also demonstrated a significantly less suppressive effect than SOCS1wt. Importantly, and supporting the specific role for nuclear expression of SOCS1, we were able to show that bronchial epithelial expression of nuclear SOCS1 was significantly increased in asthmatic patients. Further studies will be required to understand the specific mechanism of how nuclear SOCS1 suppresses virus-induced interferon induction. Our present studies clearly identify suppression of virus-induced interferon induction as a novel function of nuclear SOCS1 and, independent of its previous known nuclear function, SOCS box mediated proteasomal degradation.

In summary, our data provide novel findings relating to the requirement of nuclear SOCS1 to exert novel effects of suppression of virus-induced interferon induction. The data further demonstrate nuclear SOCS1 plays an important role in regulation of interferon deficiency in patients with AA, providing a mechanistic explanation for this important phenomenon. Because SOCS1 inhibits both virus- and interferon-induced interferon induction through distinct mechanisms, this makes inhibition of SOCS1 an attractive therapeutic target capable of restoring deficient interferon responses. The ability of SOCS1 to enhance proinflammatory responses adds further attractiveness to SOCS1 as a therapeutic target. Thus these studies provide evidence for SOCS1 as a novel therapeutic target for asthma exacerbations, a major unmet medical need.

**Key messages**

• Increased SOCS1 levels in cells from asthmatic patients impair interferon induction in asthmatic patients through its nuclear localization. However, SOCS1 did not impair virus-induced inflammatory mediators, showing specificity for antiviral immunity.
• This represents a novel mechanism explaining interferon deficiency in asthmatic patients, shows a new nuclear function of SOCS1, and identifies SOCS1 as an important therapeutic target for asthma exacerbations.

## Figures and Tables

**Table E1:** Clinical characteristics of NANA healthy control adults and adults with mild-to-moderate AA used for bronchial biopsies

Clinical characteristics	NANA subjects	Patients with AA	*P* value
No.	14	17	NA
Sex	43% M/57% F	76% M/24% F	
Age (y)	40 (24-55)	34 (21-51)	.6196
ACQ score	0	0.6 (0-3)	NA
ICS (% of patients)	0	71	NA
ICS and LABA (% of patients)	0	53	NA
Exacerbations/year	0	1 (1-4)	NA
Oral steroid courses/year (n)	0	0 (0-1)	NA
PEF (% predicted)	99 (85-123)	91 (66-123)	.1901
FEV_1_ (% predicted)	95 (80-107)	89 (56-133)	.4625
FEV_1_/FVC ratio (%)	88 (78-95)	76 (67-91)	.0002
PC_20_ histamine (mg/mL)	≥16 (NA)	1 (0-4)	.0002
Total serum IgE (U/mL)	9.5 (2-64)	127.0 (23-970)	<.0001
SPT (no. of positive responses)	0 (0-0)	4 (1-6)	NA

Values are presented as medians (ranges), except for sex, ICS, and ICS plus LABA. Statistical differences were calculated by using the Mann-Whitney *U* test. *ACQ*, Asthma control questionnaire; *F*, female; *FVC*, forced vital capacity; *ICS*, inhaled corticosteroids; *LABA*, long-acting β_2_-agonists; *M*, male; *NA*, not applicable; *PEF*, peak expiratory flow; *SPT*, skin prick test.

**Table E2:** Clinical characteristics of NANA control children and atopic children with STRA used for cultured BECs

Clinical characteristics	NANA subjects	Children with STRA	*P* value
No.	11	11	
Sex	64% M/36% F	55% M/45% F	1.00
Age (y)	7 (2-15)	11 (9-15)	.11
Family history of atopy	No	Yes	NA
Total serum IgE (U/mL)	NA	374 (121-4610)	NA
No. of positive RAST results	NA	3 (0-6)	NA
Cumulative RAST score	NA	60.4 (0.03-195)	NA
Family history of asthma	No	Yes	NA
Physician's diagnosis of asthma	No	Yes	NA
FEV_1_ (% predicted)	NA	79 (36-108)	NA
FVC (% predicted)	NA	80.6 (19-111)	NA

Values are presented as medians (ranges), except for sex. RASTs include house dust mite, mixed grass pollen, dog, cat, egg white, peanut, mixed tree pollen, *Aspergillus fumigatus*, *Alternaria* species, *Cladosporium herbarum*, and cockroach. The Mann-Whitney *U* test was used to determine significant differences between the 2 groups. The cumulative RAST score is defined as the total sum of positive RAST scores for each allergen. One hundred percent of children with STRA reported a family history of atopy and asthma and had a physician's diagnosis of asthma. These data were previously reported by Edwards et al.^E6^ *F*, Female; *FVC*, forced vital capacity; *M*, male; *NA*, not applicable.

**Table E3:** TaqMan real-time RT-PCR primers and probes used for quantitative PCR

Name	Sequence (5′-3′)
hIFN-β forward (300 nmol/L)	cgccgcattgaccatcta
hIFN-β reverse (900 nmol/L)	ttagccaggaggttctcaacaatagtgtca
hIFN-β probe (FAM 5′-TAMRA 3′ [100 nmol/L])	tcagacaacattcatctagcactggctgga
hIFN-λ1/IL-29 forward (300 nmol/L)	ggacgccttggaagagtcact
hIFN-λ1/IL-29 reverse (900 nmol/L)	agaagcctcaggtcccaattc
hIFN-λ1/IL-29 probe (FAM 5′-TAMRA 3′ [100 nmol/L])	agttgcagctctcctgtcttccccg
hIFN-λ2/3/IL-28AB forward (300 nmol/L)	ctgccacatagcccagttca
hIFN-λ2/3/IL-28AB reverse (900 nmol/L)	agaagcgactcttctaaggcatctt
hIFN-λ2/3 probe (FAM 5′-TAMRA 3′ [100 nmol/L])	tctccacaggagctgcaggccttta
hSOCS1 forward (900 nmol/L)	ccctggttgttgtagcagctt
hSOCS1 reverse (900 nmol/L)	ggtttgtgcaaagatactgggtatat
hSOCS1 probe (FAM 5′-TAMRA 3′ [100 nmol/L])	aggtaggaggtgcgagttcaggtcctg
hSOCS3 forward (900 nmol/L)	tgggacgatagcaaccacaa
hSOCS3 reverse (900 nmol/L)	cgaagtgtcccctgtttgga
hSOCS3 probe (FAM 5′-TAMRA 3′ [100 nmol/L])	tggattctccttcaattcctcagcttccc
mSOCS1 forward (900 nmol/L)	ccgtgggtcgcgagaac
mSOCS1 reverse (900 nmol/L)	aaggaactcaggtagtcacggagta
mSOCS1 probe (FAM 5′-TAMRA 3′ [100 nmol/L])	tggcgcgcatccctcttaaccc
mIFN-β forward (900 nmol/L)	ctccacgctgcgttcctgctgtg
mIFN-β reverse (900 nmol/L)	aaaggattgccacattgctc
mIFN-β probe (FAM 5′-TAMRA 3′ [100 nmol/L])	tcaagcagcctcttctcgat
mTNF-α forward (900M)	agcctgtagcccacgtcgta
mTNF-α reverse (900M)	ttgagatccatgccgttgg
mTNF-α probe (FAM 5′-TAMRA 3′ [100 nmol/L])	aaccaccaagtggaggagcagctgg
18S forward (300 nmol/L)	cgccgctagaggtgaaattct
18S reverse (300 nmol/L)	cattcttggcaaatgctttcg
18S probe (FAM 5′-TAMRA 3′ [100 nmol/L])	accggcgcaagacggaccaga

*h*, Human; *m*, mouse.

## Methods

### Viruses, cells, and reagents

Rhinovirus serotypes 16 and 1B were grown in Ohio HeLa cells and UV inactivated, as previously described.^E1^ In some experiments RV1B and RV16 were filtered by mean of centrifugation (at 12,000 rpm for 5 minutes) through a filter with a 30-kDa molecular weight cutoff (Amicon, Dublin, Ireland), thus producing a virus-free filtrate. RV1B used for *in vivo* purposes was concentrated and purified, as previously described.^E2^ BEAS-2B cells were maintained in RPMI 1640 medium with 10% FCS. Primary BECs (Clonetics, Wokingham, United Kingdom) from nonsmoking nonasthmatic donors were grown according to the manufacturer's recommended protocol. PolyI:C (Sigma-Aldrich, Dorset, United Kingdom) was made up at 1 mg/mL in water and stored at −80°C. Recombinant mouse IL-13 and recombinant human IL-4, IL-13, TNF-α, and IL-1β (R&D Systems, Abingdon, United Kingdom) were made up in sterile PBS containing 0.1% BSA and stored at −80°C. SOCS1-pORF and pORF control vector were purchased from InvivoGen (Nottingham, United Kingdom). The wild-type SOCS1-GFP, GFP empty vector, and SOCS1-GFP tagged mutants R172X, Q108X, and Δ6RA were used, as previously described.^E3^ The −125-bp fragment of the human IFN-β promoter in pGL3 was a kind gift from T. Fujita, and the IFN-λ1 promoter was made by cutting the 1kB human IFN-λ1 promoter^E4^ and subcloning into vector pGL3 (Promega, Madison, Wis). The minimal ISRE-containing promoters were purchased from Clontech (Saint-Germain-en-Laye, France). The minimal STAT1/2-containing promoter was purchased from Panomics (Vignate-Milano, Italy). All plasmids were grown in *Escherichia coli*, plasmid purified with Maxiprep (Qiagen, Crawley, United Kingdom), and stored in water at 1 μg/mL at −80°C.

### Patients with mild-to-moderate AA and NANA subjects

Bronchial biopsy specimens were obtained at bronchoscopy from a recent clinical study performed in our laboratory, which reported deficient rhinovirus-induced IFN-α/β responses in BAL cells from adults with AA compared with nonatopic healthy control adult subjects.^E5^ Inclusion and exclusion criteria have been reported.^E5^ Numbers of exacerbations in the last year were reported, as previously described.^E5^ Table E1 provides the clinical characteristics of the participants providing bronchial biopsy specimens for analysis in this study. All subjects provided written informed consent, and ethics approval for this study was given by St Mary's NHS Trust Research Ethics Committee (08/H0712/39 and 07/Q0403/20, Professor S. L. Johnston).

### BEC culture from children with STRA and NANA donors

BECs were obtained through bronchoscopies performed on 11 pediatric patients with STRA, as previously described.^E6^ NANA control children were recruited through the Royal Brompton Hospital and Children's Hospital in Bern, Switzerland. The control children (n = 11) had no personal or family history of asthma and no record of food allergy, rhinitis, or eczema. Use of bronchial brushings from patients with severe asthma and control children was approved by both the Royal Brompton NHS Trust (02/302, Professor A. Bush) and the Ethics Committee for the Canton of Bern and University Children's Hospital Bern, Switzerland (77/09, A/Professor N. Regamey). BECs were grown from bronchial brushings in bronchial epithelial cell growth medium, as previously described.^E6^ Table E2 provides the clinical characteristics of the participants in the severe asthma study.

### Animal models

*IFN-γ*^*−/−*^ and *SOCS1*^*−/−*^*IFN-γ*^*−/−*^ mice on a C57BL/6 background were bred and used, as previously described.^E7^ All breeding and experimentation was performed according to regulations outlined by the Home Office UK. The mice were lightly anesthetized with isoflurane and treated and infected intranasally. Mice were treated with 0.5 μg of recombinant mouse IL-13 (R&D Systems) 8 hours before challenge with a 5 × 10^6^ median tissue culture infectious dose of RV1B, as previously described.^E2^ *IFN-γ*^*−/−*^ and *IFN-γ*^*−/−*^*SOCS1*^*−/−*^ C57BL/6 mouse BAL macrophages were harvested by means of lavage and placed in RPMI medium with 10% FCS before plating.

### Treatment of cells and infection with rhinovirus

BEAS-2B cells, primary human BECs (Clonetics), and primary BECs obtained from asthmatic and nonasthmatic donors were infected with RV1B or RV16 (multiplicity of infection = 1). BECs (Clonetics) were treated with IL-4 and IL-13 at 50 ng/mL and IL-1β and TNF-α at 10 ng/mL (R&D Systems) or polyI:C diluted in BEBM medium and used at 1 μg/mL for various time points. Mouse BAL macrophages were counted, plated at 1 × 10^6^ cells per well, and infected with RV1B as above and harvested at the indicated time points.

### Transfection of human BECs with plasmid DNA and reporter assays

Confluent monolayers of BEAS-2B cells and primary human BECs in 12-well plates (Nunc, Rochester, NY) were transiently transfected with plasmid DNA and luciferase measured by using the Dual lucerifase assay (Promega), as previously described.^E8^

### SOCS1 immunohistochemistry

SOCS1- and SOCS3-positive cells were detected by using the EnVision Peroxidase staining method. After dewaxing, the sections were incubated with peroxidase-blocking solution and probed with primary rabbit anti-SOCS1 (catalog no. 18-003-43725; GenWay Biotech, San Diego, Calif) and rabbit anti-SOCS3 (catalog no. E16854; Spring Bioscience, Pleasanton, Calif) overnight at 4°C. The sections were then incubated with EnVision goat anti-rabbit antibody for 30 minutes (K4003; DAKO, Glostrup, Denmark). After washing, sections were incubated with chromogen (liquid diaminobenzidine and peroxide buffer). Slides were counterstained with hematoxylin to provide nuclear and morphologic detail and mounted. Irrelevant rabbit IgG (Sigma-Aldrich) was used for the primary layer as a negative control procedure. The slides were blinded, and the immunostaining intensity for total SOCS1 and SOCS3 in the airway epithelium was semiquantitatively scored as 0 to 3 (0 = negative, 1 = weak staining, 2 = moderate staining, and 3 = strong staining). The SOCS1 and SOCS3 nuclear staining of epithelial cells was counted from 2 to 3 bronchial biopsy specimens per subject, and the average was used for statistical analyses. The data for epithelial nuclear counts were expressed as the number of positive nuclei per 0.1 mm^2^ of epithelium.

### Confocal microscopy

BEAS-2B cells were seeded into transwell plates (Nunc) and transfected with GFP-tagged SOCS1 plasmids. After 24 hours, cells were fixed in 4% paraformaldehyde and counterstained with Evans Blue (red for both cytoplasm and nuclear) and 4′-6-diamidino-2-phenylindole dihydrochloride (blue for nuclear). The cells were observed at room temperature, and images were captured with a Zeiss LSM 510 inverted confocal microscope with LSM 510 software (Zeiss, Oberkochen, Germany).

### RNA isolation, cDNA synthesis, and TaqMan real-time PCR

Total RNA isolation, cDNA synthesis, and TaqMan PCR were performed, as previously described.^E6^ All data are presented as the copy number per 1 μL. Primer and probe sequences are presented in Table E3.

### SDS-PAGE and Western blotting

Total protein lysates were run on 16% Tris glycine polyacrylamide gels and transferred onto nitrocellulose membranes (Invitrogen, Paisley, United Kingdom), blocked in 5% skimmed milk, and probed with antibodies specific for human SOCS1 at 500 ng/mL (Millipore, Temecula, Calif) and human SOCS3 at 200 ng/mL (Santa Cruz Biotechnology, Santa Cruz, Calif). GFP-tagged SOCS1 proteins were analyzed by means of Western blotting with anti-GFP (Abcam, Cambridge, United Kingdom) at 1 μg/mL. Anti–α-tubulin (1 μg/mL, Santa Cruz Biotechnology) was used to detect α-tubulin as a load control. The secondary antibody used was goat anti-mouse horseradish peroxidase at 0.08 μg/mL (Santa Cruz Biotechnology). Blots were developed with ECL (GE Healthcare, Fairfield, Conn).

### ELISA

ELISAs for mouse IFN-α, IFN-λ, KC/CXCL1, RANTES/CCL5, and LIX/CXCL5 were from R&D Systems and used according to the manufacturer's recommended protocol.

### Statistical analysis

Nonclinical data are represented as means ± SEMs. At least 4 experiments were performed for *in vitro* and *ex vivo* experiments. For *in vivo* experiments, 3 experiments were performed with 4 to 7 mice per group. Time-course data in BECs and mice were analyzed by using 2-way ANOVA at a 95% CI with the Bonferroni multiple comparison test. Reporter assay and *ex vivo* BAL macrophage data were analyzed by using 1-way ANOVA at a 95% CI if significant, and differences were pinpointed with the Bonferroni multiple comparison test. Clinical data are represented as medians and were analyzed by using the Mann-Whitney *U* test. Correlations in clinical data were analyzed by using the Spearman test. A *P* value of less than .05 was considered statistically different.
